# Relationship among motives for alcohol and caffeine consumption, personality traits, and cognitive emotion regulation strategies in nonproblem alcohol drinkers

**DOI:** 10.3389/fnut.2026.1778625

**Published:** 2026-03-24

**Authors:** Naoki Kato, Shunji Oshima, Katsumi Watanabe

**Affiliations:** 1 Core Technology Laboratories, Asahi Quality & Innovations, Ltd., Ibaraki, Japan; 2 Faculty of Science and Engineering, Waseda University, Tokyo, Japan; 3 Sustainable Technology Laboratories, Asahi Quality & Innovations, Ltd., Ibaraki, Japan

**Keywords:** alcohol consumption, caffeine consumption, cognitive emotion regulation strategy, nonproblem drinker, personality trait

## Abstract

**Background:**

Alcohol and caffeine have distinct pharmacological effects, yet they are often consumed in overlapping everyday contexts (e.g., social gatherings and shared leisure time). The psychological factors that may contribute to such overlap remain unclear. This study explores associations between motives for alcohol and caffeine consumption.

**Methods:**

This study obtained Internet survey data from nonproblem alcohol drinkers in Japan aged 20–39 years (*N* = 325). Correlation analysis was conducted between the scores for the Drinking Motives Questionnaire-Revised (DMQ-R) and the Motives for Caffeine Consumption Questionnaire (MCCQ). Additionally, the study conducted an exploratory, person-centered cluster analysis based on DMQ-R and MCCQ subscale scores to descriptively summarize combined motive patterns, and compared external variables, including subscale scores from the Ten Item Personality Inventory (TIPI) and the Cognitive Emotion Regulation Questionnaire (CERQ), between the resulting clusters.

**Results:**

Among the significant positive correlation coefficients between the subscale scores for the DMQ-R and MCCQ, the largest was between conformity (DMQ-R) and social (MCCQ). Exploratory cluster analysis yielded two motive-profile clusters. The cluster characterized by relatively higher social-motive profiles showed lower neuroticism and higher openness (TIPI), as well as lower scores on several CERQ subscales (self-blame, acceptance, rumination, putting into perspective, and catastrophizing).

**Conclusion:**

The findings suggest that motives for alcohol and caffeine consumption may be associated, particularly with respect to social-context motives, and that personality traits and cognitive emotion regulation strategies may be associated with individual differences in these motive patterns.

## 1 Introduction

Alcoholic and caffeinated beverages are widely consumed in daily life, both at home and outside the home. Despite their distinct pharmacological properties, people often consume them in overlapping social and everyday contexts. Clarifying whether motives for alcohol and caffeine consumption covary—especially for social-context motives—may help generate hypotheses for future research on beverage co-use. Although motives for alcohol and caffeine consumption may not overlap across all domains, motives related to social context may show cross-beverage overlap. Therefore, investigating the relationship between motives for alcohol and caffeine consumption is meaningful.

First, regarding alcohol, Cooper et al. (1) summarized the results of 28 large-sample studies (*N* ≥ 390) and reported that social and coping motives were essentially unrelated and strongly related to problem drinking, respectively. In addition, Littlefield et al. (2) proposed that changes in neuroticism and impulsivity were associated with changes in problem drinking in early and young adulthood and that coping motives mediated this relationship. Furthermore, Hussong (3) conducted a study on American university students and found that drinking motives mediate the relationship between extroversion/neuroticism and alcohol use. Alternatively, Beninsig et al. (4) focused on university students in the Philippines and highlighted that drinking motives were directly related to extroversion/neuroticism. However, the authors found that drinking motives exerted no mediating effect on the relationship between these two personality traits and alcohol consumption. These results demonstrated the complex relationship among motives for alcohol consumption, alcohol use, and personality traits, and pointed to the effect of cultural differences. Regarding psychological scales of motives for alcohol consumption, Cooper (5) developed the Drinking Motives Questionnaire-Revised (DMQ-R), which has been used by studies abroad, while Okada (6) developed the Japanese version (DMQ-RJ). The DMQ-R was originally intended for adolescents, but later research reported its applicability to older adults (7). This scale is composed of four factors, which are a combination of two dimensions, namely, (a) avoiding negative incentives versus pursuing positive ones and (b) internally focused/directed toward oneself versus externally focused/directed toward socially significant others. The four factors are defined as follows: enhancement: positive reinforcement and self; social: positive reinforcement and social; coping: negative reinforcement and self; and conformity: negative reinforcement and social (1).

Second, for caffeine, previous studies explored the relationship between caffeine consumption and personality traits. For example, Landrum (8) reported a positive correlation between extraversion and caffeine consumption in college students, while Gurpegui et al. (9) reported that novelty seeking was associated with heavy caffeine consumption. However, knowledge on the relationship between motives for caffeine consumption and personality traits remains lacking. Regarding psychological scales of motives for caffeine consumption, the Caffeine Motives Questionnaire (CMQ) and the Motives for Caffeine Consumption Questionnaire (MCCQ) are well known (10, 11). While the CMQ has a four-factor structure, the MCCQ has the following six-factor structure composed of habit, alertness, mood, social, taste, and symptom management. The MCCQ has also been developed in Turkish, and its factor structure is the same as that of the original scale (12). However, the original and Turkish versions of the MCCQ are basically intended for young participants (mainly university students), and no Japanese version of the MCCQ has been developed.

Based on these studies, the current study explores whether motives for alcohol and caffeine consumption are associated, and whether these motives are associated with similar personality traits. This question is examined particularly with respect to social-context motives, given that alcohol and caffeine are often consumed in overlapping social contexts, without assuming that location alone determines consumption motives. In a previous study, Cooper et al. (1) examined the similarities and differences between motives for marijuana and tobacco use and alcohol consumption based on the motivational model of alcohol use by Cox and Klinger (13–15). However, the authors did not mention the relationship between motives for alcohol and caffeine consumption. In addition, studies have been conducted on the development of scales to measure motives for the consumption of alcohol mixed with energy drinks and caffeinated alcoholic beverages (16, 17). However, knowledge on the relationship between motives for alcohol and caffeine consumption or on the mediating factors remains limited.

In exploring the relationship between motives for alcohol and caffeine consumption, considering emotion regulation strategies in addition to personality traits is worthwhile. Emotion regulation pertains to the effort to influence emotions in a manner that is deemed to increase the likelihood that emotions will be beneficial, which includes alcohol consumption (18). Scholars report on the association of alexithymia, which is characterized by difficulty identifying and describing one’s emotions and an externally oriented thinking style, and is often associated with negative affect (19, 20), to increased caffeine and alcohol intake and emotional eating (21). In addition, the literature points out that emotion dysregulation can predict coping drinking motives, alcohol problems, and heavy drinking (22). Furthermore, other scholars propose that maladaptive cognitive emotion regulation strategies are associated with alcohol-related problems in oneself and romantic partners (23, 24). These studies support the association between emotion regulation strategies and alcohol and caffeine use. However, the data are mainly derived from respondents who fall under the category of problem drinkers.

In Japan, where the present study was conducted, Okada (6) examined the relationship between drinking motives and problematic alcohol use and reported that coping and enhancement motives showed the strongest associations with Alcohol Use Disorders Identification Test (AUDIT) scores. In contrast, motives related to social context (social and conformity) were reported to show weaker or no direct associations with AUDIT scores. Because the present study focuses on potential overlap in social-context motives between alcohol and caffeine consumption, we restricted our analytic sample to nonproblem drinkers screened for low-risk drinking (AUDIT scores <8) to examine motive associations within a lower-risk subgroup and improve interpretability.

This study aims to explore the relationship between motives for alcohol and caffeine consumption among nonproblem alcohol drinkers. To the best of our knowledge, this study is the first to examine this relationship using the DMQ-R and MCCQ. Given that alcoholic and caffeinated beverages are consumed in overlapping social contexts, this study hypothesizes that the subscale scores of the DMQ-R and MCCQ related to social context would be related. In addition, if a relationship exists between the subscale scores of the two questionnaires, then obtaining the background of the relationship between these motives may be possible by classifying individuals into clusters according to motive patterns and examining the differences between clusters in terms of personality traits and cognitive emotion regulation strategies. Therefore, this study emphasizes not only the relationship between motives for alcohol and caffeine consumption but also the relationship among the patterns of these motives within individuals, personality traits, and cognitive emotion regulation strategies in nonproblem alcohol drinkers.

## 2 Materials and methods

### 2.1 Participants and procedure

The researchers conducted an Internet survey with funding from Asahi Quality & Innovations, Ltd. The participants were recruited from the survey panels of a major Internet survey company in Japan. The recruitment period for this study spanned from July 9, 2024 to July 31, 2024.

First, the participants were briefed about the objective and procedure of the study, and informed consent was obtained online. They then completed the demographic survey, which requested for the following information: gender, age, marriage, cohabitants/cohabiting families, occupation, and occupation of family members. A total of 1,530 participants aged 20–59 years responded to the questionnaires described in Measures (*M*_age_ = 40.55, *SD* = 9.96; female: 56.4%; age range/distribution: 20s: 15.9%, 30s: 33.6%, 40s: 28.1%, 50s: 22.4%). Their occupations and those of their family members should not fall under the food and beverage, food service, mass media, and marketing research industries.

Specifically, however, the target of the data analysis was limited to respondents in their 20s and 30s who were screened for low-risk drinking (AUDIT <8) for two reasons. First, the average age of the participants in the original and Turkish versions of the MCCQ was 27.84 (*SD* = 10.6) and 21.74 (*SD* = 6.15) years, respectively. Second, to examine associations between alcohol and caffeine consumption motives while reducing heterogeneity related to higher-risk drinking patterns, the present study focused on young adults screened for low-risk drinking (AUDIT <8). As a result, data analysis for this study was conducted on 652 people with AUDIT scores <8 (*M*_age_ = 32.03, *SD* = 5.18; female: 55.83%; age group/distribution: 20s: 31.75%, 30s: 68.25%; Supplementary Table S1). However, partial correlation and cluster analyses regarding motives for alcohol and caffeine consumption were limited to 325 individuals with AUDIT scores <8 and who had consumed alcoholic and caffeinated beverages for at least 1 day within the past 30 days (*M*_age_ = 32.38, *SD* = 5.19; female: 52.31%, age group/distribution: 20s: 27.69%, 30s: 72.31%). In addition, for comparison with the original MCCQ development study (10), the confirmatory factor and reliability analyses of the Japanese version of the MCCQ were conducted using multiple analytic samples that included the 652-participant sample as a subset (see Section 2.3 for details). The analytic samples, sample sizes, and rationales for each restriction are summarized in Supplementary Table S2.

The participants who completed all items were sent an Amazon gift certificate worth 2,000 yen as a reward. The Ethics Committee of User Life Science, Ltd. approved the protocol of the study (reference number: ULS 202406).

### 2.2 Measures

#### 2.2.1 Alcohol Use Disorders Identification Test

AUDIT is a screening instrument developed by the WHO; the Japanese version was developed by Hiro (25). AUDIT consists of 10 questions with a full score of 40 points. Following established guidance, we used a cut-off score of 8 to indicate elevated risk for hazardous/harmful drinking. Participants scoring 8 or above were excluded from the main analytic sample (26).

#### 2.2.2 Frequency of alcohol and caffeine consumption

Participants self-reported, for each beverage type, (a) the total number of days consumed in the past 30 days (overall days) and (b) the number of days consumed alone (solitary days). We additionally derived a “with-others days” variable as overall minus solitary (values below zero, if any, were set to zero). No negative values were observed.

#### 2.2.3 Japanese version of the Drinking Motives Questionnaire-Revised (DMQ-RJ)

To measure the potential motivation to drink alcoholic beverages, the participants completed the DMQ-RJ (5, 6). This scale consists of 20 items with the following subscales: social, coping, enhancement, and conformity. The items were rated using a five‑point Likert scale (from 0 = *Almost Never*/*Never* to 4 = *Almost always*/*Always*). The participants who do not typically drink alcoholic beverages at all were asked to select 0 = *Almost Never*/*Never*.

#### 2.2.4 Japanese version of the Motives for Caffeine Consumption Questionnaire (MCCQ-J)

To measure the potential motivation to drink caffeinated beverages, the participants completed the MCCQ-J. To obtain the Japanese version, the current authors obtained permission from the original authors and applied the back-translation method to the translation process. Translation and back-translation were outsourced to a contractor, and the current authors verified the results. The original version consists of 23 items with six subscales, namely, habit, alertness, mood, social, taste, and symptom management (10). The items were rated using a five‑point Likert scale (from 1 = *Never*/*Almost never* to 5 = *Almost every time*/*Always*). The participants who do not typically consume caffeinated beverages were asked to select 1 = *Never*/*Almost never*.

#### 2.2.5 Self-perceived sensitivity/tolerance to alcohol/caffeine

To measure participants’ self-perceived sensitivity/tolerance to alcohol and caffeine (commonly understood in Japan as being “strong” or “weak”), the participants provided the most applicable responses to the following questions: “In terms of your perceived sensitivity/tolerance to alcohol/caffeine, please select the response that closely matches your perception” (from 1 = *Very weak* to 5 = *Very strong*). The participants who never consumed alcoholic or caffeinated beverages were provided with a separate option: “Never had a drink.” Out of the 757 participants in their 20s and 30s, 40 never consumed alcoholic beverages, 8 never consumed caffeinated beverages, and 4 never consumed both beverages. Participants were instructed to rate themselves toward the “sensitive/weak” end if even small amounts of caffeine tended to cause symptoms such as palpitations, gastrointestinal discomfort, or sleep disruption.

#### 2.2.6 Revised Japanese version of the Cognitive Emotion Regulation Questionnaire (CERQ-short-RJ)

To measure cognitive emotion regulation strategies, the participants completed the CERQ-short-RJ (27) [see also CERQ (28), CERQ-short (29), and CERQ-J (30)]. The short version consists of 18 items with nine subscales, namely, self-blame, other-blame, rumination, catastrophizing, positive refocusing, refocus on planning, positive reappraisal, putting into perspective, and acceptance. The items were rated using a five-point Likert scale (from 1 = *(almost) never* to 5 = *(almost) always*).

#### 2.2.7 Japanese version of the Ten Item Personality Inventory (TIPI-J)

To measure the Big Five personality traits, the participants completed the Japanese version of the TIPI-J (31, 32). The scale consists of 10 items and five subscales, namely, neuroticism, extraversion, openness, agreeableness, and conscientiousness. The items were rated using a seven-point Likert scale (1 = *Disagree strongly* to 7 = *Agree strongly*).

### 2.3 Statistical analysis

Data were analyzed using IBM SPSS Statistics version 29.0.1. We reported two-sided *p*-values and effect sizes. To control for multiple testing across the many correlational and group-comparison tests, we applied the Benjamini–Hochberg false discovery rate (BH-FDR) procedure within each table (*q* < 0.05). Results surviving BH-FDR correction are indicated in the tables (bold). The interpretation of the absolute value of the effect size was based on the classification of Mizumoto and Takeuchi (33). Correlation coefficients of 0.50 or more and Cohen’s *d* of 0.80 or more are interpreted as large.

To investigate the factor structure of the MCCQ-J, the study used AMOS version 29.0 in addition to SPSS. First, we conducted confirmatory factor analysis (CFA) and examined the fit indices for the six-factor structure which is the same as the original English version. For CFA, the data from 757 people in their 20s and 30s were compared with data from 673 of the 757 people in their 20s and 30s who consumed caffeinated beverages for at least 1 day in the past 30 days or data from 574 of the 757 people in their 20s and 30s who consumed caffeinated beverages for at least 1 day in the past 30 days and had an AUDIT score of less than 8. Rationales for each restriction are summarized in Supplementary Table S2. For the interpretation of the fit indices, we referred to the cutoff values established by Hu and Bentler (34) (cutoff values close to 0.95 for TLI and CFI, 0.08 for SRMR, and 0.06 for RMSEA). Exploratory factor analysis (EFA) was also conducted using principal axis factoring as the extraction method and Promax rotation (Kaiser normalization). Factor retention was evaluated using eigenvalues >1 and inspection of the scree plot (Supplementary Figure S1). Following Costello and Osborne (35), we evaluated simple structure based on salient pattern coefficients and potential cross-loadings. Reliability analysis using Cronbach’s alpha was used to examine the internal consistency of the scale.

To evaluate differences in the risk of hazardous/harmful drinking by gender, age, and gender–age, AUDIT scores were assessed using the Chi-square test (Supplementary Table S1). Regarding gender difference, the frequency of consumption of alcoholic/caffeinated beverages, the self-perceived sensitivity/tolerance to alcohol/caffeine, and the subscale scores of the DMQ-RJ, MCCQ-J, TIPI-J, and CERQ-short-RJ were assessed using Welch’s *t*-test (Supplementary Table S3).

To investigate the relationship among age, the item/subscale scores of the DMQ-RJ, MCCQ-J, TIPI-J, and CERQ-short-RJ, the frequency of alcohol/caffeine consumption, and the self-perceived sensitivity/tolerance to alcohol/caffeine, the study calculated Pearson’s correlation coefficients (Supplementary Tables S4, S5) and partial correlation coefficients.

In addition to variable-centered correlational analyses, we conducted an exploratory, person-centered cluster analysis to descriptively summarize combined alcohol and caffeine motive patterns. This cluster analysis was not intended as a primary hypothesis-testing approach; rather, it was used to provide an interpretable profile-based complement to the correlational findings. For each scale, we first *Z*-standardized item responses within person. We then computed within-person standardized subscale scores by using the *Z*-scored items belonging to each subscale, and these standardized subscale scores were used as clustering indicators. This approach allows interpretation of subscale scores relative to the participant’s overall response pattern within the same scale, minimizing the influence of absolute alcohol or caffeine consumption frequency. We conducted hierarchical cluster analysis using the Ward method to calculate the distance between clusters and obtained a dendrogram to analyze the range of clusters. The final number of clusters was based on interpretability and then evaluated robustness across alternative clustering solutions by comparing cluster assignments from the Ward solution with those obtained using k-means clustering. Agreement between the two solutions was quantified using Cohen’s κ derived from a cross-tabulation of cluster memberships. We additionally evaluated cluster stability using the average silhouette coefficient for the selected solution. Because clusters were defined by DMQ-RJ/MCCQ-J motive indicators, differences in these indicators across clusters were reported descriptively; inferential tests (Chi-squared test and Welch’s *t*-test) were used for external variables not used to form the clusters (e.g., demographics, TIPI-J, and CERQ-short-RJ).

## 3 Results

### 3.1 Factor structure of the MCCQ-J

As there was no Japanese version of the MCCQ to measure the motive for consuming caffeinated beverages, the authors have provisionally adapted the Japanese version of MCCQ (MCCQ-J) through forward-backward translation.

To confirm the six-factor structure of the MCCQ-J, CFA was conducted using data from 673 people in their 20s and 30s who consumed caffeinated beverages for at least 1 day in the past 30 days following the original English version of the MCCQ (10). However, the fit indices were χ^2^ = 1862.66 (*df* = 215, *p* < 0.001), CFI = 0.866, TLI = 0.842, RMSEA = 0.107, SRMR = 0.073 (Table 1), and the model fit was poor compared with that of the English version (χ^2^ = 566.9, CFI = 0.912, TLI = 0.896, RMSEA = 0.074, SRMR = 0.053). The majority of the fit indices in the current study failed to meet the cutoff points described in Statistical Analysis (34); thus, exploratory factor analysis was conducted. The Kaiser criterion suggested a three-factor solution, and the scree plot was examined (Supplementary Figure S1). The Promax-rotated pattern matrix (Supplementary Table S6) indicated limited simple structure for several items (items 9, 12, 15, 20, and 22), and the resulting factors reduced interpretability because items conceptually aligned with the original social and symptom management tended to load together in factor 1. We then evaluated a three-factor CFA; however, the three-factor model did not improve global fit relative to the provisional six-factor model (Table 1). Therefore, we retained the original six-factor scoring only as a provisional scoring framework to facilitate comparison with prior MCCQ work, while explicitly acknowledging the risk of construct misspecification and interpreting MCCQ-J subscale-level findings as exploratory.

**Table 1:** Fit indices of CFA results of the MCCQ-J.

Model	χ^2^	CFI	TLI	SRMR	RMSEA
(1) Six-factor structure (*N* = 757)	2059.26 (*df* = 215, *p* < 0.001)	0.875	0.853	0.069	0.107
(2) Six-factor structure (*N* = 673)	1862.66 (*df* = 215, *p* < 0.001)	0.866	0.842	0.073	0.107
(3) Six-factor structure (*N* = 574)	1613.39 (*df* = 215, *p* < 0.001)	0.864	0.840	0.073	0.107
(4) Three-factor structure (*N* = 673)	2331.50 (*df* = 227, *p* < 0.001)	0.828	0.809	0.093	0.117

(1): Participants in their 20s and 30s (including participants who did not consume any caffeinated beverages in the past 30 days and participants with an AUDIT score of 8 or higher). (2) and (4): Participants in their 20s and 30s who consumed caffeinated beverages at least 1 day in the past 30 days (including participants with an AUDIT score of 8 or higher). (3): Participants in their 20s and 30s who consumed caffeinated beverages at least 1 day in the past 30 days and had an AUDIT score of less than 8.

Consequently, the alpha coefficients for the MCCQ-J in the six-factor structure were comparable with those of the English version (10). Table 2 presents the results of the reliability analysis of the six subscales of the MCCQ-J, as measured using Cronbach’s alpha. It was reported that the Cronbach’s alpha values of the English version were 0.81, 0.95, 0.86, 0.91, 0.91, and 0.66 for the habit, alertness, mood, social, taste, and symptom management subscales, respectively, while the current study obtained 0.68, 0.93, 0.87, 0.92, 0.86, and 0.74, respectively for the MCCQ-J.

**Table 2:** Reliability analysis of the MCCQ-J.

Subscales/items	Participants in their 20s and 30s					Participants in their 20s and 30s who consumed caffeinated beverages at least 1 day in the past 30 days				
	(*N* = 757)					(*N* = 673)				
	*M* (*SD*)	Factor loadings	Corrected correlation	Alpha when item was deleted	Cronbach’s alpha coefficients	*M* (*SD*)	Factor loadings	Corrected correlation	Alpha when item was deleted	Cronbach’s alpha coefficients
Habit	2.96 (1.29)				0.73	3.14 (1.21)				0.68
1. …because it is a ritual for me	3.10 (1.48)	0.69	0.58			3.28 (1.41)	0.63	0.51		
8. …because it is an enjoyable habit	2.83 (1.42)	0.84	0.58			2.99 (1.38)	0.81	0.51		
Alertness	2.58 (1.11)				0.94	2.70 (1.07)				0.93
2. …because it helps when I am tired	2.59 (1.36)	0.80	0.78	0.93		2.71 (1.33)	0.78	0.75	0.92	
4. …because it helps me to concentrate	2.70 (1.37)	0.81	0.80	0.93		2.82 (1.34)	0.80	0.79	0.92	
9. …because it makes me feel that I am full of energy	2.29 (1.30)	0.83	0.78	0.93		2.38 (1.29)	0.82	0.76	0.92	
11. …because it helps me stay awake	2.69 (1.42)	0,71	0.73	0.93		2.81 (1.40)	0.69	0.71	0.92	
13. …because I become refreshed	3.17 (1.39)	0.74	0.68	0.93		3.32 (1.32)	0.71	0.64	0.93	
16. …because it helps me to wake up	2.73 (1.42)	0.76	0.78	0.93		2.86 (1.40)	0.74	0.77	0.92	
18. …because it stimulates me	2.36 (1.34)	0.81	0.79	0.93		2.47 (1.34)	0.79	0.77	0.92	
20. …because it invigorates me	2.45 (1.34)	0.85	0.80	0.93		2.58 (1.33)	0.84	0.78	0.92	
22. …because I feel physically and mentally fitter	2.27 (1.32)	0.78	0.71	0.93		2.36 (1.32)	0.76	0.69	0.92	
Mood	2.72 (1.31)				0.88	2.85 (1.27)				0.87
6. …because it improves my mood	2.70 (1.38)	0.87	0.79			2.83 (1.34)	0.85	0.77		
12. …because my mood becomes better	2.74 (1.39)	0.91	0.79			2.88 (1.36)	0.90	0.77		
Social	1.91 (1.02)				0.92	1.98 (1.02)				0.92
7. …because everyone in my company drinks it	1.83 (1.16)	0.76	0.73	0.91		1.89 (1.18)	0.75	0.72	0.91	
10. …because drinking coffee (or other caffeinated beverages) is important in social situations	1.89 (1.18)	0.83	0.79	0.91		1.94 (1.19)	0.84	0.80	0.90	
14. …because drinking coffee (or other caffeinated beverages) is a social event	1.88 (1.22)	0.84	0.81	0.90		1.94 (1.24)	0.84	0.80	0.90	
15. …because it is a pleasant addition to a good conversation	2.20 (1.30)	0.78	0.74	0.91		2.30 (1.30)	0.77	0.74	0.91	
19. …because it brings me together with other people	1.70 (1.07)	0.85	0.81	0.91		1.75 (1.08)	0.85	0.81	0.90	
21. …because it feels good with a conversation in company	1.99 (1.23)	0.84	0.80	0.91		2.06 (1.24)	0.83	0.79	0.90	
Taste	3.45 (1.34)				0.88	3.63 (1.23)				0.86
3. …because I like its taste	3.40 (1.42)	0.89	0.78			3.59 (1.31)	0.88	0.76		
17. …because it is delicious	3.49 (1.42)	0.88	0.78			3.68 (1.32)	0.87	0.76		
Symptom management	1.64 (0.95)				0.74	1.69 (0.97)				0.74
5. …because it reduces headaches	1.62 (1.07)	0.69	0.59			1.67 (1.09)	0.68	0.59		
23. …because it is good for my blood pressure	1.66 (1.06)	0.86	0.59			1.71 (1.08)	0.86	0.59		

### 3.2 Relationships between motives for alcohol and caffeine consumption

Data analysis was conducted on nonproblem drinkers in their 20s and 30s, but it was suggested that gender and age were related to motives for alcohol and caffeine consumption (Supplementary Tables S3, S4). Therefore, to investigate whether or not a relationship exists between motives for alcohol and caffeine consumption while controlling for gender and age, the study performed partial correlation analysis between the frequencies of alcohol and caffeine consumption and the subscale scores for the DMQ-RJ and MCCQ-J with gender and age as the control variables. The results indicated that the correlation coefficients between the frequencies of alcohol and caffeine consumption did not exceed 0.50. Alternatively, among the correlation coefficients between the subscale scores for the DMQ-RJ and MCCQ-J, only the correlation coefficient between conformity and social, respectively, exceeded 0.50 (Table 3).

**Table 3:** Partial correlations with gender and age as the control variables between frequencies of alcohol/caffeine consumption and the subscale scores of the DMQ-RJ and MCCQ-J.

Variable	*M* (*SD*)	1	2	3	4	5	6	7	8	9	10	11	12	13	14	15	16
Frequency of drinking alcoholic beverages																	
1. Overall days	4.16 (7.55)	―															
2. Solitary days	2.85 (6.53)	**0.84**	―														
3. With-others days	1.31 (4.06)	**0.51**	−0.04	―													
Frequency of drinking caffeinated beverages																	
4. Overall days	17.61 (11.97)	**0.22**	**0.15**	**0.16**	―												
5. Solitary days	15.64 (12.02)	**0.24**	**0.19**	**0.13**	**0.89**	―											
6. With-others days	1.96 (5.50)	−0.05	**−0.09**	0.05	**0.22**	**−0.25**	―										
DMQ-RJ																	
7. Social	11.72 (6.38)	**0.28**	**0.23**	**0.14**	0.07	**0.09**	−0.04	―									
8. Coping	10.23 (5.96)	**0.34**	**0.31**	**0.14**	**0.08**	**0.09**	−0.02	**0.63**	―								
9. Enhancement	11.52 (6.33)	**0.38**	**0.32**	**0.20**	**0.12**	**0.14**	−0.05	**0.77**	**0.81**	―							
10. Conformity	8.17 (4.72)	**0.17**	**0.18**	0.02	0.06	**0.08**	−0.05	**0.56**	**0.56**	**0.54**	―						
MCCQ-J																	
11. Habit	2.92 (1.31)	**0.17**	**0.12**	**0.11**	**0.55**	**0.51**	**0.08**	**0.23**	**0.23**	**0.26**	**0.14**	―					
12. Alertness	2.53 (1.12)	**0.17**	**0.16**	0.05	**0.34**	**0.34**	−0.01	**0.41**	**0.46**	**0.45**	**0.31**	**0.65**	―				
13. Mood	2.68 (1.32)	**0.15**	**0.13**	0.08	**0.31**	**0.32**	−0.04	**0.35**	**0.39**	**0.40**	**0.25**	**0.69**	**0.81**	―			
14. Social	1.84 (0.98)	**0.21**	**0.21**	0.05	**0.18**	**0.16**	0.04	**0.46**	**0.50**	**0.44**	**0.52**	**0.44**	**0.66**	**0.56**	―		
15. Taste	3.44 (1.35)	**0.15**	**0.12**	0.08	**0.42**	**0.41**	0.02	**0.17**	**0.17**	**0.23**	0.06	**0.74**	**0.54**	**0.63**	**0.31**	―	
16. Symptom management	1.58 (0.91)	**0.12**	**0.14**	−0.01	**0.20**	**0.18**	0.05	**0.27**	**0.38**	**0.27**	**0.39**	**0.34**	**0.56**	**0.43**	**0.69**	**0.19**	―

Participants in their 20s and 30s with AUDIT score <8 (including participants who did not consume any alcoholic/caffeinated beverages in the past 30 days). *N* = 652. Bold indicates results surviving BH-FDR correction within this table (*q* < 0.05).

Furthermore, analysis was limited to participants who consumed alcoholic and caffeinated beverages for at least 1 day each in the past 30 days, and the partial correlation coefficient was calculated with gender, age, and the self-perceived sensitivity/tolerance to alcohol/caffeine as the control variables. We observed that the only correlation coefficient between the subscale scores for the DMQ-RJ and MCCQ-J that exceeded 0.50 was between conformity and social, respectively. In contrast, the only correlation coefficient between the subscale scores for the DMQ-RJ and MCCQ-J whose *q*-value exceeded 0.05 was between conformity and taste, respectively (Table 4). As a sensitivity analysis, we repeated the partial correlation analysis using the MCCQ-J scores based on the EFA-derived three-factor solution (Supplementary Table S6). The overall pattern was similar with six-factor structure case, and only the correlation coefficient between conformity (DMQ-RJ) and factor 1 of the MCCQ-J exceeded 0.50 (Supplementary Table S7), supporting the robustness of the main correlational finding. The correlation coefficients exceeded 0.50 between the items that compose the conformity and social subscales of the DMQ-RJ and MCCQ-J, respectively. These items are “to fit in with a group you like,” “to be liked,” and “so you won’t feel left out” for conformity and “because it brings me together with other people” for social (Table 5). Based on these results, the study found evidence of an association suggesting that among nonproblem drinkers in Japan in their 20s and 30s, motives for alcohol and caffeine consumption may be related, which is particularly the case for motives related to a sense of belonging.

**Table 4:** Partial correlations with gender, age, and the self-perceived sensitivity/tolerance to alcohol/caffeine as the control variables between the DMQ-RJ and MCCQ-J subscales.

MCCQ-J	DMQ-RJ			
	Social	Coping	Enhancement	Conformity
Habit	**0.21**	**0.26**	**0.27**	**0.16**
Alertness	**0.37**	**0.49**	**0.43**	**0.30**
Mood	**0.29**	**0.45**	**0.44**	**0.24**
Social	**0.41**	**0.47**	**0.36**	**0.55**
Taste	**0.14**	**0.22**	**0.27**	0.05
Symptom management	**0.20**	**0.36**	**0.18**	**0.40**

Participants in their 20s and 30s with AUDIT score <8 who had consumed alcoholic and caffeinated beverages for at least 1 day each in the past 30 days. *N* = 325. Bold indicates results surviving BH-FDR correction within this table (*q* < 0.05).

**Table 5:** Partial correlations with gender, age, and the self-perceived sensitivity/tolerance to alcohol/caffeine as the control variables between the DMQ-RJ and MCCQ-J items.

Item	*M* (*SD*)	1	2	3	4	5	6	7	8	9	10	11	12	13	14	15	16
DMQ-RJ social																	
1. Because it helps you enjoy a party.	3.10 (1.36)	―															
2. To be sociable.	2.77 (1.37)	**0.77**	―														
3. Because it makes social gatherings more fun.	2.92 (1.36)	**0.73**	**0.82**	―													
4. Because it improves parties and celebrations.	2.91 (1.34)	**0.70**	**0.77**	**0.84**	―												
5. To celebrate a special occasion with friends.	2.86 (1.32)	**0.51**	**0.53**	**0.62**	**0.63**	―											
DMQ-RJ conformity																	
6. Because your friends pressure you to drink.	1.85 (1.23)	**0.28**	**0.38**	**0.32**	**0.38**	**0.36**	―										
7. So that others will not kid you about not drinking.	1.77 (1.14)	**0.28**	**0.38**	**0.33**	**0.37**	**0.33**	**0.85**	―									
8. To fit in with a group you like.	2.01 (1.22)	**0.40**	**0.52**	**0.50**	**0.50**	**0.42**	**0.65**	**0.65**	―								
9. To be liked.	1.85 (1.07)	**0.34**	**0.49**	**0.44**	**0.44**	**0.36**	**0.66**	**0.67**	**0.82**	―							
10. So you will not feel left out.	1.80 (1.08)	**0.32**	**0.43**	**0.39**	**0.39**	**0.32**	**0.69**	**0.68**	**0.78**	**0.85**	―						
MCCQ-J social																	
11. Because everyone in my company drinks it	1.98 (1.23)	**0.19**	**0.24**	**0.26**	**0.26**	**0.27**	**0.32**	**0.29**	**0.41**	**0.40**	**0.37**	―					
12. Because drinking coffee (or other caffeinated beverages) is important in social situations	2.06 (1.26)	**0.31**	**0.40**	**0.38**	**0.36**	**0.35**	**0.41**	**0.36**	**0.44**	**0.47**	**0.43**	**0.61**	―				
13. Because drinking coffee (or other caffeinated beverages) is a social event	2.03 (1.32)	**0.22**	**0.32**	**0.29**	**0.28**	**0.30**	**0.42**	**0.39**	**0.46**	**0.49**	**0.45**	**0.60**	**0.76**	―			
14. Because it is a pleasant addition to a good conversation	2.47 (1.37)	**0.24**	**0.34**	**0.36**	**0.32**	**0.39**	**0.30**	**0.26**	**0.39**	**0.41**	**0.38**	**0.57**	**0.61**	**0.64**	―		
15. Because it brings me together with other people	1.82 (1.13)	**0.19**	**0.29**	**0.26**	**0.25**	**0.26**	**0.48**	**0.44**	**0.54**	**0.53**	**0.50**	**0.62**	**0.66**	**0.71**	**0.61**	―	
16. Because it feels good with a conversation in company	2.21 (1.31)	**0.22**	**0.33**	**0.32**	**0.27**	**0.34**	**0.39**	**0.33**	**0.46**	**0.45**	**0.40**	**0.54**	**0.59**	**0.66**	**0.72**	**0.69**	―

Participants in their 20s and 30s with AUDIT score <8 who had consumed alcoholic and caffeinated beverages for at least 1 day each in the past 30 days. *N* = 325. Bold indicates results surviving BH-FDR correction within this table (*q* < 0.05). The items in the table are the expressions in the English version.

In addition, prior to investigating the relationship among the patterns of these motives, personality traits, and cognitive emotion regulation strategies, the study examined the correlation coefficients between the subscale scores of the TIPI-J or CERQ-short-RJ and those of the DMQ-RJ or MCCQ-J. None of the correlation coefficients between the subscale scores of the TIPI-J or CERQ-short-RJ and the DMQ-RJ or MCCQ-J exceeded 0.50. Specifically, the highest correlation coefficients between the scales were as follows: between openness (TIPI-J) and conformity (DMQ-RJ): 0.28, between openness (TIPI-J) and habit (MCCQ-J): 0.23, between catastrophizing (CERQ-short-RJ) and coping (DMQ-RJ): 0.32, and between positive reappraisal (CERQ-short-RJ) and social (MCCQ-J): 0.42 (Supplementary Table S8). Furthermore, in the partial correlation analysis between the subscale scores of the DMQ-RJ and MCCQ-J (control variables: gender, age, the self-perceived sensitivity/tolerance to alcohol/caffeine, and the subscale scores of the TIPI-J and CERQ-short-RJ), none of the correlation coefficients exceeded 0.50. Nevertheless, the analysis again observed a significant positive correlation between conformity (DMQ-RJ) and social (MCCQ-J; *r* = 0.46, *q* < 0.05; Supplementary Table S9).

### 3.3 Comparison of the patterns of the DMQ-RJ and MCCQ-J for personality traits and cognitive emotion regulation strategies

To investigate combined patterns of alcohol and caffeine consumption motives, the study conducted a cluster analysis using the subscale scores of the DMQ-RJ and MCCQ-J and then examined between-cluster differences in external variables (e.g., personality traits and cognitive emotion regulation strategies). Given that the frequency of alcohol/caffeine consumption was related to the subscale scores of the DMQ-RJ and MCCQ-J (Table 3), the standardized subscale scores for the 20 items of the DMQ-RJ and the 23 items of the MCCQ-J for each participant were used for cluster analysis (see Section 2.3 for details). Excluding participants whose *Z*-scores were not calculated, the remaining 307 participants were divided into two motive-profile clusters (Table 6). The two-cluster solution showed moderate agreement across clustering algorithms (Ward vs. k-means; Cohen’s κ = 0.562, *p* < 0.001). As a supplementary index of cluster separation, a two-step cluster analysis with the number of clusters fixed at two yielded a fair average silhouette value (mean silhouette = 0.30), supporting a descriptively interpretable two-cluster summary.

**Table 6:** Characteristics of each cluster defined by DMQ-RJ/MCCQ-J motive indicators.

Variable	Cluster 1 (*N* = 168; 52.9% female)	Cluster 2 (*N* = 139; 52.6% female)	*t*-test (*df*)	*p* (uncorrected)	*q* (BH-FDR)	Cohen’s *d*	95% CI
	*M* (*SD*)						
DMQ-RJ (*Z*-score)							
Social	2.37 (2.93)	0.45 (2.66)					
Coping	−2.09 (1.83)	2.39 (1.86)					
Enhancement	1.66 (2.69)	2.05 (2.29)					
Conformity	−1.94 (2.36)	−4.88 (2.03)					
MCCQ-J (*Z*-score)							
Habit	0.49 (0.68)	0.39 (0.70)					
Alertness	0.11 (0.33)	0.26 (0.27)					
Mood	0.21 (0.59)	0.35 (0.56)					
Social	−0.43 (0.47)	−0.58 (0.45)					
Taste	0.90 (0.86)	0.82 (0.66)					
Symptom management	−0.80 (0.72)	−0.98 (0.74)					
Age	32.35 (5.16)	32.61 (5.06)	−0.44 (286.96)	0.660	0.822	−0.05	[−1.42, 0.90]
Frequency of drinking alcoholic beverages							
Overall days	7.37 (8.91)	9.47 (9.35)	−2.00 (277.15)	0.047	0.135	−0.23	[−4.18, −0.03]
Solitary days	5.05 (8.39)	6.82 (8.55)	−1.81 (281.45)	0.072	0.183	−0.21	[−3.69, 0.16]
With-others days	2.32 (5.09)	2.65 (5.86)	−0.53 (261.85)	0.597	0.822	−0.06	[−1.59, 0.92]
Frequency of drinking caffeinated beverages							
Overall days	20.82 (10.42)	21.10 (9.89)	−0.24 (291.08)	0.813	0.822	−0.03	[−2.57, 2.02]
Solitary days	18.91 (10.78)	19.40 (10.67)	−0.39 (285.68)	0.695	0.822	−0.05	[−2.91, 1.95]
With-others days	1.91 (4.78)	1.70 (4.84)	0.38 (282.45)	0.707	0.822	0.04	[−0.88, 1.30]
Self-perceived sensitivity/tolerance to							
Alcoholic beverages	3.03 (1.02)	3.11 (1.02)	−0.60 (284.55)	0.547	0.822	−0.07	[−0.30, 0.16]
Caffeinated beverages	3.48 (0.90)	3.46 (0.96)	0.22 (274.45)	0.822	0.822	0.03	[−0.19, 0.24]
TIPI-J							
Extraversion	7.20 (3.07)	6.95 (2.91)	0.72 (291.23)	0.471	0.774	0.08	[−0.43, 0.92]
Agreeableness	9.51 (2.51)	9.44 (2.52)	0.23 (283.70)	0.815	0.822	0.03	[−0.50, 0.64]
Conscientiousness	8.07 (2.73)	7.48 (3.20)	1.70 (258.68)	0.091	0.210	0.20	[−0.09, 1.27]
Neuroticism	8.11 (2.63)	9.02 (2.93)	−2.83 (267.19)	0.005	**0.019**	−0.33	[−1.55, −0.28]
Openness	7.36 (2.64)	6.61 (2.67)	2.46 (283.11)	0.014	**0.047**	0.28	[0.15, 1.36]
CERQ-short-RJ							
Self-blame	6.30 (1.83)	7.11 (1.76)	−3.88 (289.69)	*p* < 0.001	**0.001**	−0.44	[−1.21, −0.39]
Acceptance	6.63 (1.83)	7.30 (1.71)	−3.29 (293.27)	0.001	**0.006**	−0.38	[−1.07, −0.27]
Rumination	6.41 (1.84)	7.23 (1.78)	−3.93 (288.91)	*p* < 0.001	**0.001**	−0.45	[−1.23, −0.41]
Positive refocusing	5.89 (2.07)	6.19 (2.18)	−1.21 (276.29)	0.228	0.436	−0.14	[−0.78, 0.19]
Refocus on planning	6.73 (1.87)	6.97 (1.93)	−1.10 (280.02)	0.274	0.485	−0.13	[−0.67, 0.19]
Positive reappraisal	5.93 (2.05)	6.01 (2.20)	−0.31 (273.48)	0.756	0.822	−0.04	[−0.56, 0.41]
Putting into perspective	5.63 (1.92)	6.32 (1.83)	−3.23 (290.74)	0.001	**0.006**	−0.37	[−1.12, −0.27]
Catastrophizing	6.06 (1.80)	6.81 (1.83)	−3.61 (281.97)	*p* < 0.001	**0.003**	−0.42	[−1.17, −0.34]
Other-blame	5.07 (1.77)	5.45 (2.15)	−1.64 (252.70)	0.103	0.215	−0.19	[−0.83, 0.08]

Participants in their 20s and 30s with AUDIT score <8 who had consumed alcoholic and caffeinated beverages for at least 1 day each in the past 30 days. Participants with zero within-scale variance (i.e., identical responses across all items within a scale, yielding *SD* = 0) were excluded from the within-person *Z*-score computation and subsequent clustering (*n* = 18). Clusters were derived from DMQ-RJ/MCCQ-J motive indicators; therefore, differences in these indicators across clusters are descriptive of the clustering solution. For between-cluster comparisons of external variables (not used to derive the clusters), bold indicates results surviving BH-FDR correction across tests in this table (*q* < 0.05).

For the comparison of gender between clusters, the results of the Chi-squared test were χ^2^_(1)_ = 0.002, *p* = 0.966, and Cramer’s *V* = 0.002. The results of Welch’s *t*-test between the clusters showed no BH-FDR-significant differences in age, the frequencies of alcohol and caffeine consumption, and the self-perceived sensitivity/tolerance to alcohol/caffeine (all *q* > 0.05). Because clusters were defined by DMQ-RJ/MCCQ-J motive indicators, we described the two clusters’ motive profiles descriptively. For the comparison of external variables, Welch’s *t*-tests indicated that Cluster 1 showed lower neuroticism and higher openness (TIPI), as well as lower scores on several CERQ subscales (self-blame, acceptance, rumination, putting into perspective, and catastrophizing) than Cluster 2; these differences remained significant after BH-FDR correction (all *q* < 0.05).

## 4 Discussion

Although alcohol and caffeine have distinct pharmacological effects, they may be consumed in partially overlapping everyday and social contexts. However, the psychological factors that may contribute to this overlap remain unclear. To address this concern, this study examined the relationship between motives for alcohol and caffeine consumption using the DMQ-RJ and MCCQ-J, respectively. However, because the MCCQ-J factor structure should be regarded as provisional in the present dataset, interpretations at the subscale level are exploratory. Therefore, we regard the following observed associations between alcohol and caffeine consumption motives as exploratory and need further study regarding the MCCQ-J.

The results of the correlation analysis between the subscale scores for the DMQ-RJ and MCCQ-J were consistent with the study hypothesis that motives for alcohol and caffeine consumption related to social context may be associated with one another. Among the significant positive correlations between the subscale scores of the DMQ-RJ and MCCQ-J, the largest correlation coefficient that was the only one to exceed 0.50 was that between conformity (DMQ-RJ) and social (MCCQ-J). Furthermore, correlation analysis between the items for conformity (DMQ-RJ) and social (MCCQ-J) demonstrated that the items related to the sense of belonging were particularly strongly positively correlated. Although the MCCQ includes internally oriented motives such as mood improvement and alertness, these items primarily reflect regulation of everyday affective or arousal states rather than high intensity coping with distress. In contrast, coping motives in the DMQ-R capture negative reinforcement processes that have been shown to be more strongly associated with problematic alcohol use (1, 6). Thus, internally oriented motives may differ in functional intensity across substances, which may partly explain why externally oriented social motives showed clearer cross-beverage convergence in the present nonproblem sample. In addition, although not a study that excluded problem drinkers, other scholars report that, among the subscales of the Caffeinated Alcoholic Beverages Motives Questionnaire (CABMQ), the strongest correlation with conformity (DMQ-R) was found for conformity (CABMQ) (17). Importantly, the CABMQ focuses on motives for consuming caffeinated alcoholic beverages within the same drinking occasion, that is, simultaneous co-use of alcohol and caffeine. In contrast, the present study did not assume simultaneous consumption; nevertheless, we observed an association between social context motives for alcohol and caffeine consumption (MCCQ-J social and DMQ-RJ conformity). This suggests that convergence in social context motives may extend beyond situations of concurrent intake and instead reflect shared social functions across beverages with distinct pharmacological properties.

Furthermore, to explore the background of the relationship between motives for alcohol and caffeine consumption, this study focused on personality traits and cognitive emotion regulation strategies, conducted cluster analysis using the subscale scores of the DMQ-RJ and MCCQ-J, and compared the subscale scores of the TIPI-J and CERQ-short-RJ between clusters. The results suggested that nonproblem drinkers in their 20s and 30s who drink alcohol and caffeine for at least 1 day each in the past 30 days may be descriptively grouped largely by the level of their social motives for alcohol and caffeine consumption. Furthermore, people who consumed alcohol and caffeine for social motives displayed significantly lower neuroticism and higher openness (TIPI), as well as lower scores on several CERQ subscales (self-blame, acceptance, rumination, putting into perspective, and catastrophizing). Thus, the current study found that personality traits and cognitive emotion regulation strategies were associated with patterns of motives for alcohol and caffeine consumption. Importantly, the study performed partial correlation analysis with gender, age, the self-perceived sensitivity/tolerance to alcohol/caffeine, and the subscale scores of the TIPI-J and CERQ-short-RJ as the control variables, and observed the significant positive correlation between conformity (DMQ-RJ) and social (MCCQ-J; *r* = 0.46, *q* < 0.05; Supplementary Table S9). This finding indicated that other factors are also involved in this relationship. For example, one potential factor is a sense of belonging, which can be measured using the Collective Self-Esteem Scale (36). Alternatively, preference for ambient social cues (e.g., music) may be a relevant factor in social-context motives across beverages. Thus, investigating individual differences in sensory and contextual preferences (taste, smell, and sound) may help clarify variability in social-context motives.

## 5 Limitations and further research

The first limitation of this study is the restricted analytic sample. Several analyses were restricted to nonproblem drinkers (AUDIT <8) and participants who reported alcohol and/or caffeinated beverage consumption within the past 30 days (Supplementary Table S2). These restrictions may influence observed effect sizes. For example, excluding problem drinkers may reduce variability in coping-related drinking motives, potentially altering associations compared with samples that include problem drinkers. Accordingly, the present findings are most generalizable to young adult nonproblem drinkers who recently consumed alcohol and caffeinated beverages, and may not generalize to problem drinkers, infrequent consumers, or other age groups.

The second limitation concerns the exploratory cluster analysis. Because cluster membership is defined by DMQ-RJ/MCCQ-J motive indicators, between-cluster differences in these motive indicators are inherently expected and should be interpreted descriptively rather than inferentially.

The third limitation is that it was conducted only on Japanese respondents. When considering the complex relationship among motives for alcohol and caffeine consumption, alcohol use, and personality traits (3, 4), as well as the differences in personality traits between countries (37, 38), a possibility exists that the relationship between the subscale scores of the DMQ-R and MCCQ identified in this study among nonproblem drinkers in Japan may not be identified among non-Japanese individuals.

The fourth pertains to the model fit of the MCCQ-J. Given the suboptimal CFA fit and the imperfect simple structure of the alternative EFA solution, the MCCQ-J subscale scores may be subject to construct misspecification in the present dataset. Accordingly, we use the six-factor scoring only provisionally and treat all MCCQ-J subscale-level interpretations as exploratory. The results of the original version demonstrated that the subscale scores differ according to gender, age, and the type of caffeine beverage consumed (10). The participants in the current study were older on average than those of the English version. In addition, green tea is a typical beverage in Japanese culture. Therefore, future studies should examine the relationship between these factors and the low fit indices of the MCCQ-J. For example, in the original version of the scale, participants were asked to think of every caffeinated product they consume and answer accordingly, but it might be meaningful to compare the results of asking them to think of just one type of beverage, such as coffee, green tea, black tea, or energy drinks.

The fifth limitation is that the tendency to use each emotion regulation strategy was only examined as a general tendency using the CERQ-short-RJ, which assesses individuals’ typical responses to negative or unpleasant events. In the present study, we did not measure momentary negative affect, situational context, or regulation awareness during episodes of alcohol or caffeine use. As a result, no direct data were obtained on whether people consciously use alcohol and caffeinated beverages differently in relation to the motives and cognitive emotion regulation strategies. Accordingly, differences in CERQ scores observed in the study should not be interpreted as reflecting reduced regulatory awareness or capacity; rather, they may reflect differences in affect activation or contextual exposure. Importantly, in the research on emotion regulation, a view exists that the adaptability of strategies should be determined by individual contexts. Moreover, a number of scholars report that one’s perception of a certain context moderates the relationship between cognitive emotion regulation strategies and psychological health (30, 39–41). Although the current study did not focus on the adaptability of strategies or psychological health, investigating the people’s perception of certain situations and considering emotion regulation strategies prior to alcohol or caffeine consumption could contribute to an in-depth understanding of the relationship between motives for alcohol and caffeine consumption.

The sixth limitation is that alcohol and caffeine use were assessed primarily as the number of days of consumption within the past 30 days to maintain a consistent behavioral index across beverages. Although alcohol quantity per occasion was assessed only categorically via an AUDIT item, this provides a coarse indicator of typical quantity, and we did not obtain finer quantitative amount measures that would be more suitable for correlational analyses. We also did not assess caffeine dose per occasion or the frequency of consuming caffeinated alcoholic beverages. Therefore, we could not examine whether motive patterns differ by amount per occasion, nor could we determine whether alcohol and caffeine were consumed simultaneously on the same occasion. Future studies should incorporate episode-level quantitative measures of alcohol and caffeine amount and explicit assessments of co-use to test whether motive patterns vary by dose and context.

## 6 Conclusion

In this exploratory analysis of young adults screened for low-risk drinking (AUDIT <8), we identified an association between motives for alcohol and caffeine consumption and found that personality traits and cognitive emotion regulation strategies may be associated with the differences in the patterns of these motives. This exploratory study contributes to the development of research that elucidates the complex relationship between alcohol and caffeine consumption and to understanding how these beverages may relate to emotion regulation strategies.

## Data Availability

The raw data supporting the conclusions of this article will be made available by the authors, without undue reservation.
