# Correction: Relationship among motives for alcohol and caffeine consumption, personality traits, and cognitive emotion regulation strategies in nonproblem alcohol drinkers

**DOI:** 10.3389/fnut.2026.2012574

**Published:** 2026-10-12

**Authors:** Naoki Kato, Shunji Oshima, Katsumi Watanabe

**Affiliations:** 1 Core Technology Laboratories, Asahi Quality & Innovations, Ltd., Ibaraki, Japan; 2 Faculty of Science and Engineering, Waseda University, Tokyo, Japan; 3 Sustainable Technology Laboratories, Asahi Quality & Innovations, Ltd., Ibaraki, Japan

**Keywords:** alcohol consumption, caffeine consumption, cognitive emotion regulation strategy, nonproblem drinker, personality trait

The reference for citation 29 was erroneously written as:

Garnefski N, Kraaij V. Relationships between cognitive emotion regulation strategies and depressive symptoms: a comparative study of five specific samples. *Pers Individ Dif* . (2006) 40:1659–69. doi: 10.1016/j.paid.2005.12.009

It should be:

Garnefski N, Kraaij V. Cognitive emotion regulation questionnaire – development of a short 18-item version (CERQ-short). *Pers Individ Dif* . (2006) 41:1045–53. doi: 10.1016/j.paid.2006.04.010

The original version of this article has been updated.

